# Selected Papers from the Third International Symposium on Life Science

**DOI:** 10.3390/md18020117

**Published:** 2020-02-18

**Authors:** Valentin A. Stonik

**Affiliations:** 1 G.B. Elyakov Pacific Institute of Bioorganic Chemistry, Far Eastern Branch, Russian Academy of Sciences, 690022 Vladivostok, Russia; stonik@piboc.dvo.ru; 2 School of Natural Sciences, Far East Federal University, 690001 Vladivostok, Russia

The search for and isolation of marine biologically active compounds, as well as relevant studies on their structure and properties are important for the adding knowledge about molecular diversity in nature and creation of medicines and other useful products on this basis. Long-term studies by G. B. Elyakov Pacific Institute of Bioorganic Chemistry (PIBOC) belonging to the Russian Academy of Sciences have led to the isolation and structural elucidation of many hundreds of new marine natural compounds as well as to creation of a series of marine drugs, food additives, and functional food ingredients which were approved for industrial production and medicinal or other applications in Russia. PIBOC actively collaborates with scientists from many countries of the world and performs regular marine expeditions in the North-Western Pacific and other geographic areas using R/V “Academik Oparin”. Colleagues from other countries, particularly from Vietnam, also participate in these expeditions. Over the course of its more than 50-year history, our institute has repeatedly organized international scientific conferences.

The Third International Symposium on Life Science, held September 4–8 in Vladivostok, has been also organized by PIBOC. Scientists from several towns of the Russian Federation from Moscow to Vladivostok, as well as chemists and biologists from Germany, the Republic of Korea, the Peoples Republic of China, and Taiwan attended this Symposium and delivered 81 plenary lectures, and oral and poster presentations co-authored by scientists from several other countries. A sub-symposium of the Korus series (Korean-Russian symposiums, started from 2012) was held in the frameworks of this scientific meeting. The aim the Third Symposium on Life Science was to share advanced ideas not only in the field of chemistry of marine natural products, but also in determination of molecular mechanisms of action of new marine metabolites, pharmacology, enzymology, and molecular genetics in order to achieve important new scientific results. 

The Special Issue “Selected Papers from the Third International Symposium on Life Science (http:/mdpi.com/journal/marinedrugs/special_issues/Selected Papers from the Third International Symposium on Life Science) in the open access journal Marine Drugs (ISSN 1660-3397) was running from the end of 2018 to the end of 2019. Totally, it comprises 17 articles, concerning with different aspects of Life Science and recent experimental studies, carried out on the basis of marine natural products.

Structures and biological activities of low molecular weight secondary metabolites from marine organisms were discussed in several selected papers. Tabakmakher et al. from PIBOC and colleagues from University Medical Center Hamburg-Eppendorf, Germany, and Institute of Marine Biochemistry, Vietnam, have described seven new polysulfated sterols isolated from the Vietnamese marine sponge *Halichondria vansoesti*. Of particular interest is the fact that compounds similar to some steroids isolated by this group which contain both bromine and chlorine atoms have never been found among marine steroids. The effects of these compounds on human prostate cancer cells, expression of the prostate-specific antigen (PSA), and glucose uptake have been discussed. This was the first report on the ability of marine steroids to suppress PSA expression/androgen receptor signaling in cancer cells [1]. 

A series of oxysterols including 4 previously unknown compounds has been isolated by Kolesnikova et al. from extracts of a sponge *Inflatella* sp. collected in the Sea of Okhotsk [2]. The influence of isolated compounds on the viability and reactive oxygen species (ROS) formation in neuronal Neuro2a cells using 6-hydroxydopamine-induced cell model of Parkinson’s disease was clarified. Some compounds of this series showed the essential neuroprotective activity in these in vitro experiments, probably due ROS scavenging effect.

Yurchenko et al. from PIBOC together with Vietnamese colleagues from Institute of Technology Research and Application (Nhatrang) and Graduate University of Science and Technology (Hanoi) have isolated and studied a new melatonin analogue, 6-hydroxy-*N*-acetyl-β-oxotryptamine, from the marine-derived fungus *Penicillium* sp. KMM 4672 and several other compounds of different chemical nature from *Aspergillus flocculosis* and *Aspergillus* sp. Activities of these metabolites in the 6-hydroxydopamine- and paraquat-induced Parkinson’s disease cell models were studied. The new melatonin analogue protects Neuro2a cells more effectively in these experiments in comparison with melatonin itself [3].

Ga-Bin Park et al. from Kosin and Jnje Universities (Republic of Korea) have discussed interesting activities of gliotoxin, a mycotoxin, containing disulfide bond in a piperazine ring and an aromatic amino acid residue and isolated from the marine fungus *Aspergillus fumigatus.* Its action on paclitaxel-resistant ovarian cancer cells has been studied. They have shown that treatment with gliotoxin at nanomolar concentrations inhibits growth and reduces resistance to cytotoxic agents in these cancer cells. Gliotoxin induces apoptotic cell death in an autophagy-dependent manner via the death-associated protein kinase-1 (DAPK1)-TAp63 signaling pathway. The treatment with gliotoxin before paclitaxel treatment inhibited the expression of multidrug resistant-associated proteins and increases expression of DAPK1 and TAp63. The obtained results suggest that DAPK1-mediated TAp63 upregulation is one of the critical pathways inducing apoptosis in chemoresistant cancer cells [4].

The possible microbial origin of some highly active marine metabolites, such as toxins and antitumor agents isolated from invertebrates, has previously been the subject of discussions. Indeed, it has been found that many marine invertebrates contain endo- and epibiotic microorganisms and that some metabolites found in marine invertebrates are structurally related to bacterial natural products. This suggests the microbial origin of some marine invertebrate metabolites [5]. Moreover, microbial origin of bryostatins in bryozoans and some other natural products in lithistid sponges was confirmed by experiments [6]. 

In this Issue, Makarieva et al. (PIBOC) have reported isolation of more than 20 bacterial strains from the secreted mucus trapping of polychaete *Chaetopterus variopedatus*, and the strain CB-1-14 was recognized as a new species belonging to the genus *Vibrio.* This bacterium was cultured, and 6-*epi*-monanchorin A was obtained from both cells and culture broth using preparative HPLC. This natural compound along with the related guanidine alkaloid monanchorin were earlier found only in marine sponges and the same polychaete species. Thus, the microbial origin of this guanidine alkaloid in marine invertebrates has been established [7].

Venoms of sea anemones are well known as a rich source of peptides acting on different molecular targets such as enzymes, membrane receptors and ion channels. Magnificamide, the major α-amylase inhibitor, comprising of 44 amino acid residues (4770 Da), was isolated from the sea anemone *Heteractis magnifica* mucus. Sintsova and colleagues from PIBOC and University of Lueven, Belgium, have reported that the recombinant magnificamide inhibits porcine and human saliva α-amylases in low nanomolar concentrations and could be considered as a promising drug candidate for the type 2 diabetes treatment [8]. 

A series of articles has been published by Korean scientists, in majority from National mitochondrial signaling of cardiovascular and metabolic disease center (Pusan, Republic of Korea) and their colleagues from other Korean Universities with participation of Russian co-authors from PIBOC. Professor Jin Park is scientific leader of many such studies. They have investigated molecular mechanisms of action of the Russian medicine Histochrome and active substance of this drug, echinochrome A (Ech A). In its two drug forms, Histochrome has been permitted for clinical application in cardiology and ophthalmology in the Russian Federation. In the published papers, other potential applications of these biopreparations and new peculiarities of their action on cellular and organism levels have been considered. 

Ga-Bin Park and co-authors have investigated a possibility to use Ech A as a well-established and non-toxic antioxidant to facilitate ex vivo application of sensitive to oxidative damage hematopoetic stem and progenitor cells (HSPCs), released from the bone morrow. Ech A promoted ex vivo expansion of peripheral blood-derived CD34^+^ cells by suppressing reactive oxygen species generation and p38 MAPK/JNK phosphorylation in them. Activation of Lyn kinase and p110δ as a mechanism to enhance expansion of CD34^+^ cells was also shown. An assumption that Ech A initially induces Src/Lyn activation, upregulates p110δ, and finally activate PI3K/Akt pathway was made. More or equal hematopoietic colony forming cells were induced by CD34^+^ cells expanded in the presence of Ech A in comparison with unexpanded CD34^+^ cells. Therefore, Ech A is an effective agent for promoting cell proliferation and maintaining the stemness of HSPCs. It is beneficial to maintain self-renewal potential of CD34^+^ cells during the ex vivo and possibly in vivo expansion of HSPCs [9].

In the related article, Jl Hye Park and collaborators has discussed the results of the studies on cell protective effect of Histochrome under oxidative stress in human cardiac progenitor cells (hCPCs). There are small portions of these stem cells in ischemic hearts, where they participate in repairing the damaged heart tissues. Histochrome does not influence surface expression markers of hCPCs. It reduces cellular and mitochondrial ROS levels in these cells at oxidative stress, induced by H_2_O_2_, protects hCPCs, and shows anti-apoptotic effects through downregulation of pro-apoptotic signals and upregulation of anti-apoptotic signals. Histochrome delayed the progression of cellular senescence in hCPCs. It was concluded that the use of histochrome as biosafe agent is promising as potential therapeutic strategy at application of patients-derived hCPCs to treat cardiovascular diseases [10]. 

In confirmation of positive effects of Ech A at cardiovascular problems, Ran Kim and co-authors have reported a significant effect of Ech A on the injured region and behavioral decline at ischemic stroke in a rat middle cerebral artery occlusion model after reperfusion. Ech A alleviated the infarcted brain region and encouraged affirmative behavioral changes after ischemic stroke. It altered the expression levels of cell viability-related factors. It was concluded that the protective role of this natural compound against cell death is connected with its antioxidant effect [11].

In their paper, Su-Jeong Oh and co-authors have discussed a beneficial impact of Ech A on inflammatory bowel disease, using a murine model of experimental colitis. Intravenous injection of this compound prevented subsequent lethality and body weight loss in colitis-induced mice. In in vitro experiments, this preparation stimulated generation of regulatory T cells, suppressed the activation of proinflammatory M1 type macrophages and induced the production of M2 type macrophages. It has been suggested that due to these features of the action, Ech A can correct imbalances in the intestinal immune system, help resolve inflammation and initiate tissue repair [12].

Unexpectedly, the Russian team from PIBOC, G.M. Somov Institute of Epidemiology and Microbiology (Vladivostok), and Institute of Vaccines and Sera (Moscow) has found that Ech A not only exhibits anti-oxidant properties, but also shows anti-viral activities against tick-borne encephalitis virus and herpes simplex virus type 1. A mixture of Ech A, ascorbic acid and tocopherol (5:5:1) showed even higher anti-oxidant and anti-viral effects in comparison with Ech A itself [13].

Not only Ech A, but also some other quinoid pigments from sea urchins, so-called spinochromes, have also been shown to be potential therapeutic agents. In fact, Chang Shin Yoon and co-authors have discussed the results of the studies on protective action of spinochrome D (spD), a structural analogue of Ech A, on cardiomyocytes against doxorubicin (Dox). As it is well known, doxorubicin demonstrates suppressive activity against different cancers, but, being cardiotoxic, it increases ROS level in heart cells. Authors of this article have reported that spD protected the Ac16 human cardiomyocyte cell line against Dox cytotoxicity, but did affect anticancer properties of DOX in relation of MCF-7 human breast tumor cells. As it was established by proteomic and metabolomic analyses, its action led to alterations in glutathione metabolism. The increase of ATP level and oxygen consumption rate, induced by spD, was detected in galactose-treated AC-16 cardiomyocytes. These findings suggested that spD could be considered as a potential cardioprotective agent at Dox therapy of oncological patients [14].

Sokolova and colleagues from PIBOC reported that Ech A is soluble in aqueous solutions of carrageenans from red algae. Its complexes with carrageenans showed the ability to decrease expression of pro-inflammatory cytokines Il-6 and TNFα and increase the expression of anti-inflammatory cytokine Il-10. Thus, carrageenans can modulate biological properties of Ech A [15].

Problems of the studies on biopolymer natural compounds and last results, obtained by participants of the symposium at investigation of the corresponding biopreparations were also discussed and later published as scientific articles involved in this issue. For example, a group of co-authors from PIBOC and Far East Federal University has published the results of mutagenesis studies and structure-function relationship of GalNac/Gal lectin and its mutant forms. This lectin, named CGL, from the mussel *Crenomytilus grayanus* (family Mytilidae, class Bivalvia) is a representative of novel lectin family with β-trefoil fold. The crystal structure of this lection and mutagenesis studies revealed three carbohydrate-binding sites capable to recognize globotriose on the surface of breast cancer cells. In their article, Kovalchuk and colleagues have analyzed how alanine substitution of His 37, His 129, Glu-75, His 85, Asn 27, and Asn 119 in these sites affects mucin-binding capability of CGL. It was shown that this binding is determined by the number of hydrogen bonds in CGL-ligand complexes [16].

The review of Besednova and coauthors “Metabolites of Seaweeds as Potential Agents for the Prevention and Therapy of Influenza Infection” (G.M. Somov Institute of Epidemiology and Microbiology) contains analysis of literature data about anti-influenza effects of algal polysaccharides such as fucoidans, carrageenans, and ulvans as well as other biopolymer substances from algae (lectins and polyphenols). It was concluded that use of recently developed drugs can lead to the selection of resistant viral strains. That is why some metabolites from algae with a broad spectrum of anti-viral activity could be of interest as a potential basis for creation of new drugs [17].

The obtaining of recombinant form of alkaline hosphatase/phosphodiesterase, Cam PhoD, from the marine bacterium *Cobetia amphilecti* KMM 296, expressed in *Escherichia coli* cells, has been described in the article of Noskova et al from PIBOC and Far East Federal University. The enzyme catalyzes the cleavage of diester and phosphate bonds in nucleotides. It was shown that Cam PhoD, exhibiting maximum activity in the presence of Co^2+^ and Fe^3+^ ions, is a new member of PhoD family [18].

Genomic studies on two recently described species of marine bacteria, *Zobellia amuskyensis* and *Zobellia laminariae* from the PIBOC Collection of marine microorganisms (KMM), have been discussed in the paper of Chernysheva et al. Two novel draft genomes were obtained and compared with known genomes of this genus representatives. Pan-genome of this genus is composed of 4853 orthologous clusters. Carbohydrate active enzyme repertoires were highly diverse and biotechnogically promising as biocatalysts for obtaining of oligosacchharides and other products with possible applications in food and pharmaceutical industries [19].

In summary, this issue covers a series of in their majority experimental studies recently carried out in the field of marine bioactive low molecular weight and biopolymer substances. In their publications, scientists from Russia, the Republic of Korea, Vietnam, Germany, and Belgium have discussed new results obtained at the search for new marine natural compounds, their isolation and structure determination, biological activity, interaction with molecular targets, biomedicinal properties, biogenesis, and perspectives of practical application. A wide spectrum of biological activities was reported for these compounds, including antitumor, neuroprotective, anti-inflammatory, antioxidant, antiviral, and other useful properties. In my opinion, the discovered new properties found for echinochrome A, the active substance of the Russian drugs belonging to the Histochrome series, are of particular interest and should open the way to new areas of medical use of this type drugs.

As a guest editor, I am thankful to all scientists from diverse research institutes and universities, who contributed to the success of a special Issue “Selected Papers from the Third International Symposium on Life Science”.

## References

[1] Tabakmakher K.M., Makarieva T.N., Denisenko V.A., Popov R.S., Dmitrenok P.S., Dyshlovoy S.A., Grebnev B.B., Bokemeyer C., von Amsberg G., Cuong N.X. (2019). New Trisulfated Steroids from the Vietnamese Marine Sponge Halichondria vansoesti and Their PSA Expression and Glucose Uptake Inhibitory Activities. Mar. Drugs.

[2] Kolesnikova S.A., Lyakhova E.G., Kalinovsky A.I., Popov R.S., Yurchenko E.A., Stonik V.A. (2018). Oxysterols from a Marine Sponge Inflatella sp. and Their Action in 6-Hydroxydopamine-Induced Cell Model of Parkinson’s Disease. Mar. Drugs.

[3] Yurchenko E.A., Menchinskaya E.S., Pislyagin E.A., Trinh P.T.H., Ivanets E.V., Smetanina O.F., Yurchenko A.N. (2018). Neuroprotective Activity of Some Marine Fungal Metabolites in the 6-Hydroxydopamin- and Paraquat-Induced Parkinson’s Disease Models. Mar. Drugs.

[4] Park G.-B., Jeong J.-Y., Kim D. (2019). Gliotoxin Enhances Autophagic Cell Death via the DAPK1-TAp63 Signaling Pathway in Paclitaxel-Resistant Ovarian Cancer Cells. Mar. Drugs.

[5] König G.M., Kehraus S., Seibert S.F., Abdel-Lateff A., Müller D. (2006). Natural products from marine organisms and their associated microbes. Chembiochem..

[6] Haygood M.G., Davidson S.K., Schmidt E.W., Faulkner D.J. (1999). Microbial Symbionts of Marine Invertebrates: Opportunities for Microbial Biotechnology. J. Mol. Microbiol. Biotechnol..

[7] Makarieva T., Shubina L., Kurilenko V., Isaeva M., Chernysheva N., Popov R., Bystritskaya E., Dmitrenok P., Stonik V. (2019). Marine Bacterium Vibrio sp. CB1-14 Produces Guanidine Alkaloid 6-epi-Monanchorin, Previously Isolated from Marine Polychaete and Sponges. Mar. Drugs.

[8] Sintsova O., Gladkikh I., Kalinovskii A., Zelepuga E., Monastyrnaya M., Kim N., Shevchenko L., Peigneur S., Tytgat J., Kozlovskaya E. (2019). Magnificamide, a β-Defensin-Like Peptide from the Mucus of the Sea Anemone Heteractis magnifica Is a Strong Inhibitor of Mammalian α-Amylases. Mar. Drugs.

[9] Park G.-B., Kim M.-J., Vasileva E.A., Mishchenko N.P., Fedoreyev S.A., Stonik V.A., Han J., Lee H.S., Kim D., Jeong J.-Y. (2019). Echinochrome A Promotes Ex Vivo Expansion of Peripheral Blood-Derived CD34+ Cells, Potentially through Downregulation of ROS Production and Activation of the Src-Lyn-p110δ Pathway. Mar. Drugs.

[10] Park J.H., Lee N.-K., Lim H.J., Mazumder S., Rethineswaran V.K., Kim Y., Jang W.B., Ji S.T., Kang S., Kim D.Y. (2019). Therapeutic Cell Protective Role of Histochrome under Oxidative Stress in Human Cardiac Progenitor Cells. Mar. Drugs.

[11] Kim R., Hur D., Kim H.K., Han J., Mishchenko N.P., Fedoreyev S.A., Stonik V.A., Chang W. (2019). Echinochrome A Attenuates Cerebral Ischemic Injury through Regulation of Cell Survival after Middle Cerebral Artery Occlusion in Rat. Mar. Drugs.

[12] Oh S.-J., Seo Y., Ahn J., Shin Y.Y., Yang J.W., Kim H.K., Han J., Mishchenko N.P., Fedoreyev S.A., Stonik V.A. (2019). Echinochrome A Reduces Colitis in Mice and Induces In Vitro Generation of Regulatory Immune Cells. Mar. Drugs.

[13] Fedoreyev S.A., Krylova N.V., Mishchenko N.P., Vasileva E.A., Pislyagin E.A., Iunikhina O.V., Lavrov V.F., Svitich O.A., Ebralidze L.K., Leonova G.N. (2018). Antiviral and Antioxidant Properties of Echinochrome A. Mar. Drugs.

[14] Yoon C.S., Kim H.K., Mishchenko N.P., Vasileva E.A., Fedoreyev S.A., Stonik V.A., Han J. (2019). Spinochrome D Attenuates Doxorubicin-Induced Cardiomyocyte Death via Improving Glutathione Metabolism and Attenuating Oxidative Stress. Mar. Drugs.

[15] Sokolova E.V., Menzorova N.I., Davydova V.N., Kuz’mich A.S., Kravchenko A.O., Mishchenko N.P., Yermak I.M. (2018). Effects of Carrageenans on Biological Properties of Echinochrome. Mar. Drugs.

[16] Kovalchuk S.N., Buinovskaya N.S., Likhatskaya G.N., Rasskazov V.A., Son O.M., Tekutyeva L.A., Balabanova L.A. (2018). Mutagenesis Studies and Structure-function Relationships for GalNAc/Gal-Specific Lectin from the Sea Mussel *Crenomytilus grayanus*. Mar. Drugs.

[17] Besednova N., Zaporozhets T., Kuznetsova T., Makarenkova I., Fedyanina L., Kryzhanovsky S., Malyarenko O., Ermakova S. (2019). Metabolites of Seaweeds as Potential Agents for the Prevention and Therapy of Influenza Infection. Mar. Drugs.

[18] Noskova Y., Likhatskaya G., Terentieva N., Son O., Tekutyeva L., Balabanova L. (2019). A Novel Alkaline Phosphatase/Phosphodiesterase, CamPhoD, from Marine Bacterium Cobetia amphilecti KMM 296. Mar. Drugs.

[19] Chernysheva N., Bystritskaya E., Stenkova A., Golovkin I., Nedashkovskaya O., Isaeva M. (2019). Comparative Genomics and CAZyme Genome Repertoires of Marine Zobellia amurskyensis KMM 3526T and Zobellia laminariae KMM 3676T. Mar. Drugs.

